# A method to improve protein subcellular localization prediction by integrating various biological data sources

**DOI:** 10.1186/1471-2105-10-S1-S43

**Published:** 2009-01-30

**Authors:** Thai Quang Tung, Doheon Lee

**Affiliations:** 1 Department of Bio & Brain Engineering, KAIST, Daejeon City, Republic of Korea

## Abstract

**Background:**

Protein subcellular localization is crucial information to elucidate protein functions. Owing to the need for large-scale genome analysis, computational method for efficiently predicting protein subcellular localization is highly required. Although many previous works have been done for this task, the problem is still challenging due to several reasons: the number of subcellular locations in practice is large; distribution of protein in locations is imbalanced, that is the number of protein in each location remarkably different; and there are many proteins located in multiple locations. Thus it is necessary to explore new features and appropriate classification methods to improve the prediction performance.

**Results:**

In this paper we propose a new predicting method which combines two key ideas: 1) Information of neighbour proteins in a probabilistic gene network is integrated to enrich the prediction features. 2) Fuzzy k-NN, a classification method based on fuzzy set theory is applied to predict protein locating in multiple sites. Experiment was conducted on a dataset consisting of 22 locations from Budding yeast proteins and significant improvement was observed.

**Conclusion:**

Our results suggest that the neighbourhood information from functional gene networks is predictive to subcellular localization. The proposed method thus can be integrated and complementary to other available prediction methods.

## Background

One of the most important goals in modern cell biology is to understand how proteins function in the context of compartments that organize them in the cellular environment. In order to answer this challenging question the very first step is to identify the subcellular localizations of proteins. Although conducting various experiments can help determine the subcellular locations of proteins, this approach is time-consuming and expensive. Recent advances in large-scale genome sequencing have resulted in the huge accumulation of proteins whose functions are unknown. Thus it has become necessary to develop automatic computational method which can identify subcellular localization fast and reliable.

During the last decade, there have been many efforts to develop computational methods to predict protein subcellular localization. Early studies [1-8] mostly based on protein transporting mechanism, that is some proteins have a small sequence called "sorting signal" which can decide where the protein can be located. Unfortunately, the protein transporting mechanism is well-understood for only a limited number of localizations and many proteins do not have a sorting signal in practice.

The second approach is to use global information of protein sequences as features of prediction systems. Amino acid compositions [9-12] and its variation feature extraction methods such as dipeptide compositions [13], gap amino acid pair compositions [14,15], pseudo-amino acid compositions [16-19] and n-dipeptide compositions [20] were introduced. A large number of classification algorithms were applied using these composition features such as Neural Networks [11], Support Vector Machines [12,14,21], k-nearest neighbours (k-NN) [13,22,23]. Despite the high accuracies reported in many works, in [19] Chou has revealed that prediction performance of this approach is very sensitive to the redundancy of dataset used for training the classification model.

In several studies [17,18], Chou and his colleagues proposed functional domain compositions as prediction features. The works were based on an assumption that proteins having overlapped sets of functional domains or protein motifs possibly share the same functional characteristics which leads to the conclusion that they belong to the same localization. Considering the number of functional domains is huge and there exist redundancy among them, Chou further introduced a more compact set of features by mapping the proteins to Gene ontology terms [19]. Gene ontology database, a controlled vocabulary used to describe the biology of gene products in any organism, was utilized to extract features from protein sequences. Each protein can be represented as a vector in GO-space whose attributes correspond to the GO terms that appear in the annotation of the protein. Many recent works [23-25] have followed this feature extraction process and yield good prediction accuracies.

Most of previously mentioned studies focused on only a small number of subcellular localizations while this number is considerable large in practice. Combining with the fact that there exist proteins localizing in multiple sites, the problem of subcellular localization prediction is very challenging. Furthermore, the distribution of protein over subcellular locations is extremely imbalanced, that make the prediction much more difficult for locations which have only a small number of training samples. Only a few efforts have been made to deal with those issues. Lee's approach [21] was to develop a particular classification algorithm called PLPD (protein localization predictor based on D-SVDD) that can efficiently handle imbalanced dataset while Chou (ISORT) [22] attempted to find novel scheme to grasp core features of proteins. In this study, aiming to develop a reliable protein subcellular localization prediction method we need to find both a good feature extraction scheme and an appropriate computational algorithm.

Previously, the subcellular localization was predicted using information extracted from the protein sequence itself. With the available sources of biological information such as microarray data, experimental protein interaction data, which help reveal protein functions, it is worth exploring such kinds of information to predict protein subcellular localization. In [26], Drawid *et al.* have shown the correlation between subcellular localizations and gene expression levels. Michelle *et al.* have pointed out in [27] that interacting proteins might be found in the same subcellular locations. Therefore, in this study, we attempt to enrich features of a protein for subcellular localization prediction by incorporating the characteristic of proteins which are considered as functionally related or have a biological interaction with the query protein. We expect that such information is predictive to subcellular localization. The functional relationships between proteins can be extracted from probabilistic functional gene networks which are constructed by integrating heterogeneous functional genomics and proteomics data.

**Figure 1:**
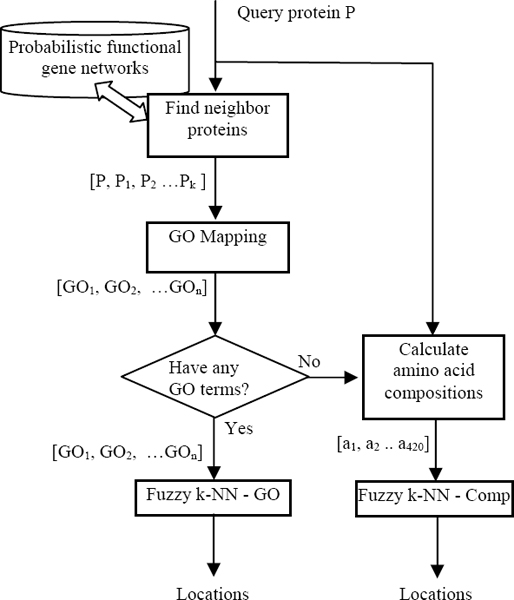
Prediction flowchart.

Figure 1 illustrates the flowchart of our proposed prediction method. Given a query protein *P*, we first find its neighbour proteins *P*_1_, *P*_2 _... *P*_*k *_from a functional gene network. The next steps are similar to those of ISORT: A GO mapping process is followed to build a vector to represent the set {*P*, *P*_1_, *P*_2_,... *P*_*k*_}. If this process fails to create a vector in GO-space, amino acid and dipeptide compositions of the query protein sequence are estimated to make a feature vector. The feature vectors are passed to corresponding classifiers to predict the protein locations. However, instead of using k-NN classifiers like ISORT, we adapted fuzzy k-NN classification models to predict subcellular locations. Not like the k-NN who assigns predicted locations by a roughly voting on locations of nearest neighbour proteins, the fuzzy k-NN estimates a membership value for each location indicating how much degree the protein can belong to the location. Thus, such fuzzy set theory based models are much more appropriated for the multi-label multi class protein subcellular localization prediction.

## Results and discussion

### Dataset

In order to evaluate the performance of the proposed method, we conduct experiments on Yeast proteins. The original dataset was published by Chou [22] containing 3555 proteins occurred in 22 subcellular locations which are considered as the largest number of labels so far. The subcellular localization information of those proteins was observed through a high throughput experiment conducted by Hur [28]. Chou has proceed a sequence alignment process to remove the sequence redundancy, that is of all the proteins, none among them have larger than 40% sequence identity which can warrant the performance validation is not biased. Among 3555 proteins, three are not listed in the SWISS-PROT [29] version 54.0 and removed from the dataset to make the final dataset consist of 3552 proteins. The protein label distribution is listed on Table 1. Among 3552 proteins, 2473 of them are known to appear in a single subcellular location. The others coexist in more than one location, in specific, 1012, 58, 8 and 1 appear in 2, 3, 4 and 5 locations respectively. Due to this 'multiplex location' feature, the number of classified protein is larger than the number of different protein and can be estimated as:

(1) $$\tilde{\text{N}}=2473+2\times 1012+3\times 58+4\times 8+5\times 1=\text{4708}$$

**Table 1:** Numbers of proteins in the dataset

Subcellular locations	Number of proteins
Mitochondrion	494
Vacuole	129
Spindle pole	58
Cell periphery	106
Punctate composite	123
Vacuolar_membrane	54
ER	272
Nuclear periphery	59
Endosome	43
Bud neck	60
Microtubule	20
Golgi	40
Late Golgi	36
Peroxisome	20
Actin	29
Nucleolus	157
Cytoplasm	1576
ER to Golgi	6
Early Golgi	51
Lipid particle	19
Nucleus	1333
Bud	23

Total number of classified proteins	4708
Total number of different proteins	3552

### Evaluation measures

To evaluate the algorithm performance, jack-knife test is employed because it is the most rigorous and objective test, besides it is simple and effective to be implemented with k-NN classifier. In the jack-knife test process, each protein is singled out in turn as a test sample, the remaining proteins are used as training set to calculate test sample nearest neighbours and predict the class.

For multi-label learning paradigms, only one measurement is not sufficient to evaluate the performance of a predictor owing to the variety of correctness in prediction. Lee [21] proposed three measurements (Measure-I, Measure-II and Measure-III) for the evaluation of a protein localization predictor. First, to check the overall success rate regarding to the total number of the unique proteins *N*, Measure-I is defined as:

(2) $$\frac{1}{N}\sum_{i=1}^{N}\phi [L(p_{i}),Y_{i}^{k}]$$

where *L*(*p*_*i*_) is the true label set of a protein *p*_*i*_, $Y_{i}^{k}$ is the predicted top-*k *labels by a predictor, and

(3) $$\phi [L(p_{i}),Y_{i}^{k}]=\{\begin{array}{ll} 1, & \text{if any label in }Y_{i}^{k}\text{ is in }L(p_{i})\\ 0, & \text{otherwise} \end{array}\;$$

Note that owing to the multi-label protein localization problem, it is not sufficient to evaluate the performance of a predictor by checking only the topmost label predicted true. Thus, we check the real label set with the predicted top-*k *labels using the *ϕ*[.,.] function. The *k *value is given by user, and we set *k *= 3 in this study since the numbers of true localization sites of most proteins are less than or equal to three.

To check the overall success rate regarding to the total number of classified proteins, $\tilde{N}$, we also evaluate the performance of a prediction system by using Measure-II:

(4) $$\frac{1}{\text{Ñ}}\sum_{i=1}^{N}\phi [L(p_{i}),Y_{i}^{k_{i}}]$$

where $Y_{i}^{k_{i}}$ is the predicted top-*k*_*i *_labels by a predictor, and the *ϕ*[.,.] function returns the number of labels which is predicted correctly. Note that the *k*_*i *_value determined by the number of true labels of a protein *p*_*i*_, not by user.

As mentioned earlier, a dataset of protein localization is imbalanced in nature. Including overall success rate, thus, the information on the success rate of each class and the average rate of the success rates of each class is useful to evaluate the performance of a predictor. To achieve this, the Measure-III was defined as:

(5) $$\frac{1}{\mu }\sum_{l=1}^{\mu }\frac{1}{{\text{ñ}}_{\text{l}}}\sum_{i=1}^{{\text{ñ}}_{\text{l}}}\Delta [Y_{i}^{k_{i}},l]$$

where *μ *= 22 is total number of labels or classes, l is a label index, ${\tilde{n}}_{l}$ is the number of proteins in the *l*^*th *^label, and

(6) $$\Delta [Y_{i}^{k_{i}},l]=\{\begin{array}{ll} 1, & \text{if any label in }Y_{i}^{k_{i}}\text{ is equal to l}\\ 0, & \text{otherwise} \end{array}$$

### Advance of fuzzy k-NN classification

In ISORT prediction system proposed by Chou, a k-NN algorithm is utilized to predict protein subcellular locations from GO-space. Given a protein with unknown location, the system will assign its labels according to labels of proteins which have highest similarity to the query protein. Since vectors representing proteins in GO-space are high-dimensional and sparse, this instance-based learning method is an appropriated selection. However, due to the nature of multisite localization of proteins, it is expected that the fuzzy k-NN method will outperform the ordinary k-NN.

In the first experiment, we analyzed the performance of fuzzy k-NN classification method and compared to those of the k-NN classification algorithm in the scope of multisite multi-class protein subcellular localization prediction. From the original dataset, we selected only 2135 proteins which can be represented in GO-space for classification. For fuzzy k-NN classifier, the number of nearest neighbours *k *is set to 25 and the fuzziness parameter m is set to 1.05. For the ordinary k-NN, the number of nearest neighbours is varying from 1 to 3, according to Chou [21]. Table 2 shows the prediction performance in three measures. For the Measure-I, different numbers of topmost labels were selected ranging from 1 to 3. The results from Table 2 clearly show that the fuzzy k-NN outperform in terms of overall prediction success rate regarding to total number of unique proteins (Measure-I) and total number of classified proteins (Measure-II). Besides, improvements are observed for most of subcellular locations, leading to the increase of Measure-III which indicates that the fuzzy k-NN is better in handling imbalanced training data.

**Table 2:** Performance comparison between fuzzy k-NN and k-NN models in three measures

	ISORT (1-N)	ISORT (2-NN)	ISORT (3-NN)	Fuzzy K-NN (*k *= 25, *m *= 1.05)
Measure I (k = 1) (%)	50.68	55.41	56.91	**62.25**

Measure I (k = 2) (%)	59.67	68.85	70.40	**79.77**

Measure I (k = 3) (%)	60.23	72.93	76.96	**86.14**

Measure II (%)	47.83	55.73	58.63	**63.52**

Mitochondrion	**43.81**	28.43	38.13	35.12
Vacuole	30.26	26.32	26.32	**31.58**
Spindle pole	27.78	16.67	22.22	**38.89**
Cell periphery	26.98	31.75	30.16	**34.92**
Punctate composite	6.56	4.92	3.28	**19.67**
Vacuolar membrane	8.11	**10.81**	0	8.11
ER	41.61	44.97	41.61	**53.02**
Nuclear periphery	**50.00**	35.00	45.00	**50.00**
Endosome	**40.74**	**40.74**	**40.74**	**40.74**
Bud neck	**36.11**	30.56	33.33	**36.11**
Microtubule	**45.46**	**45.46**	**45.46**	**45.46**
Golgi	**28.57**	**28.57**	23.81	23.81
Late Golgi	**21.74**	13.04	17.39	**21.74**
Peroxisome	**33.33**	**33.33**	**33.33**	**33.33**
Actin	**52.94**	23.53	23.53	**52.94**
Nucleolus	13.92	15.19	20.25	**32.91**
Cytoplasm	49.08	64.72	66.18	**79.88**
ER to Golgi	**100.00**	**100.00**	**100.00**	**100.00**
Early Golgi	20.00	30.00	**33.33**	26.67
Lipid particle	18.18	9.09	**27.27**	9.09
Nucleus	63.47	78.03	**83.25**	77.91
Bud	**76.92**	53.85	23.08	7.69

Measure III (%)	37.98	34.77	35.35	**39.07**

**Figure 2:**
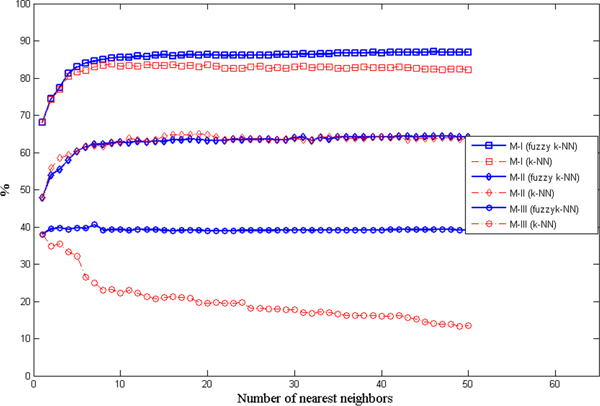
Prediction performance of k-NN and fuzzy k-NN on three measures (M-I, M-II and M-III) are plotted against the number of nearest neighbours.

In order to support the above claim, we further analyzed the prediction performance respecting to the number of nearest neighbours used for assigning labels which is varying from 1 to 50. In Figure 2, the performance in three measures is plotted against the number of nearest neighbours. As this number increases, the Measure-I and Measure-II values increase until stable states are reached. From the figure we can see that both classifiers achieve similar success rates. However, there is a big different in the behaviour of Measure-III. As the number of nearest neighbours increase, average success rate achieved by k-NN dramatically decreases, while those of fuzzy k-NN is retained as a stable value regardless the number of nearest neighbours. This implies the prediction decision made by k-NN is biased which is a foreseeable phenomenon when prediction is performed on an imbalanced dataset. To avoid such the issue, 1-NN, 2-NN or 3-NN were applied in ISORT which on the other hand achieved quite low performance on Measure-I and Measure-II, as shown on Table 2. In contrast, the fuzzy k-NN still can achieve high prediction success rates by considering more nearest neighbours while keeping the prediction decision unbiased.

### Incorporating neighbourhood information can improve prediction performance

Integrating information from neighbour proteins which are considered as functional related to query protein, we aim two goals: 1) this information is expected to be predictive to classify subcellular locations so that it can helps improve the prediction performance. 2) Increasing the prediction coverage, that is increasing the total number of proteins which can be featured in GO-space.

Among 3552 proteins in the dataset, by following the GO-mapping process proposed by Chou, there are 2135 proteins having at least one associated GO term. It makes the prediction coverage is only 60%. By applying our proposed feature extraction approach, proteins without GO annotation still might be featured in GO-space by taking those of its neighbour proteins which are considered probabilistically functional related. The total number of predictable proteins increases up to 3017, it makes 85% prediction coverage.

The performance of Fuzzy k-NN classifier in three measures is reported in Table 3 There were a slightly improvements in Measure-I and Measure-II, the best improvement was observed in Measure-II with 5.08% higher. Due to the imbalance in localization distribution, one may expect that given a protein, the possibility its neighbour proteins belonging to a major location such as "cytoplasm" or "nucleus" is high. As a result, adding such information will guide the prediction system to make biased decisions. However, as the results shown in Table 3, success rates on 14 locations was increased. It implies that the information extracted from neighbour proteins is indeed predictive to subcellular localization.

**Table 3:** A prediction performance comparison to show the effectiveness of incorporating neighbourhood information

	No NI^(*)^	NI^(*)^
Prediction coverage (%)	60	**84**

Measure-I (*k *= 3) (%)	86.14	**87.50**

Measure-II (%)	63.52	**67.76**

Mitochondrion	35.12	**57.85**
Vacuole	**31.58**	15.30
Spindle pole	38.89	**60.00**
Cell periphery	**34.92**	30.34
Punctate composite	**19.67**	16.50
Vacuolar membrane	8.11	**22.73**
ER	**53.02**	51.89
Nuclear periphery	**50.00**	48.98
Endosome	40.74	**44.12**
Bud neck	36.11	**52.73**
Microtubule	**45.46**	31.58
Golgi	23.81	**42.86**
Late Golgi	21.74	**29.03**
Peroxisome	33.33	**65.00**
Actin	52.94	**58.62**
Nucleolus	32.91	**58.62**
Cytoplasm	79.88	**80.68**
ER to Golgi	**100.00**	66.67
Early Golgi	26.67	**48.87**
Lipid particle	**9.09**	6.67
Nucleus	77.91	**80.59**
Bud	7.69	**23.81**

Measure-III (%)	39.07	**45.15**

^(*) ^**Note**: No NI: Prediction without incorporating neighbourhood information; NI: Prediction with neighbourhood information.

Although the improvements on Measure-I and Measure-II are not significantly large, we should note that the 25% of GO-space based prediction coverage increasing is an important factor to foster the performance of a complete protein subcellular localization prediction system. In such a system, the prediction based on sequence global information (amino acid compositions and its relatives) has very poor accuracies as reported in [19,21]. Thus larger number of proteins which are localization predictable with GO-space representation, the better prediction performance the system can achieve. This statement is clearly verified by results in the next experiment.

### Performance comparison

As the final experiment, we compare performance of proposed method to those of ISORT and PLPD which, to our knowledge, are considered the best prediction methods dealing with multisite multi-class protein subcellular localization prediction problem. PLPD system predicts protein subcellular localization by using a density-induced support vector data description classification model trained on a combination of features consisted of amino acid composition, dipeptide composition, gap-pair amino acid compositions and occurrences of protein motifs.

Among 3552 proteins in the original dataset, there are 2015 proteins which can be represented in GO-space. By integrating neighbourhood information, this number increases up to 3017 proteins. Fuzzy k-NN with *k *= 25 and *m *= 1.05 is used to prediction those protein locations using GO features. The remained 535 proteins without associated GO annotation are represented by vectors of amino acid compositions and dipeptide compositions. The fuzzy k-NN with *k *= 25 and *m *= 1.05 is then used to predict protein locations. The performance in three measures is reported in Table 4.

**Table 4:** Prediction performance (%) of ISORT, PLPD and proposed method

	ISORT	PLPD	Proposed method
Measure-I (*k *= 3) (%)	72.89	**85.32**	85.25

Measure-II (%)	53.84	59.26	**64.36**

Mitochondrion	32.1862	0.81	**53.4413**
Vacuole	17.8295	**20.83**	16.2791
Spindle pole	12.069	42.31	**51.7241**
Cell periphery	22.6415	21.25	**28.3019**
Punctate composite	1.626	**15.66**	13.8211
Vacuolar membrane	0	**36.36**	18.5185
ER	32.3529	1.04	**45.2206**
Nuclear periphery	20.339	5.41	**42.3729**
Endosome	25.5814	34.29	**34.8837**
Bud neck	26.6667	19.15	**48.3333**
Microtubule	25	**52.94**	30
Golgi	17.5	32.14	**40**
Late Golgi	11.1111	**33.33**	25
Peroxisome	25	33.33	**65**
Actin	17.2414	25	**58.6207**
Nucleolus	22.293	11.4	**56.051**
Cytoplasm	64.5305	**99.85**	77.7919
ER to Golgi	50	**80**	66.6667
Early Golgi	27.451	32.43	**41.1765**
Lipid particle	15.7895	**30.77**	5.2632
Nucleus	**81.6204**	65.28	79.6699
Bud	39.1304	**70.59**	21.7391

Measure-III (%)	26.7254	34.74	**41.8125**

As we can see from Table 4, the ISORT method show 72.89%, 53.84% and 26.73% according to the Measure-I, Measure-II and Measure-III, the proposed method showed 85.25%, 64.36% and 41.81% for the three performance measures respectively. This implies that the success rates of our method were 12.36, 10.52 and 15.08% higher than the ISORT method regarding the Measure-I, the Measure-II and the Measure-III, respectively.

The performance of PLPD which was reported in [21] is shown on Table 4. It shows a similar success rate with our method on Measure-I. However our method is 5.1% and 7.07% larger on Measure-II and Measure-III respectively. A further analysis of the success rates on individual classes can tell us that the proposed method can handle imbalance dataset much better than PLPD. Although PLPD predict protein in cytoplasm (1576 proteins) with almost perfect success rate (99.85%), the success rates for other major classes Nucleus (1333 proteins), Mitochondrion (494 proteins) and ER (272 proteins) are seriously degraded, the worst case is happened to Mitochondrion where the prediction results almost wrong (success rate = 0.81%). Those problematic results can be explained as the PLPD tends to predict proteins to cytoplasm location.

In conclusion, the experiment results have shown that the proposed method is superior to ISORT in terms of three performance measures. When compare to PLPD, it also achieved significant improvement in Measure-II and Measure-III. Detail analysis on individual class success rates reveal the proposed method can handle imbalanced dataset much better that PLPD.

## Conclusion

Although many previous works have been done for the task of protein subcellular localization prediction, there are only a few attempts to predict proteins which locate in multi-sites. Besides, as the number of subcellular locations is large in practice and the numbers of proteins in locations are imbalancedly distributed, it is much harder to achieve a good prediction success rate. Thus there are still rooms for researchers to develop new methods to challenge those issues.

In order to develop a reliable prediction of subcellular localization of proteins both good features for a protein and a good computational algorithm are ultimately needed. In this study we first proposed a method both to enrich the prediction features of proteins and enlarge the prediction coverage by incorporating information from other proteins which are considered as functionally related. Such relationships are extracted from a probabilistic functional gene network which was constructed by integrating heterogonous biological sources such as protein interaction databases, microarray co-expression data. The prediction features are extracted by mapping the protein and its neighbours to a vector in GO-space whose attributes corresponds to GO terms associated to them.

On the other hand, we have adapted a fuzzy k-NN classification to predict multisite multiclass protein subcellular localization. Experiments were conducted on an imbalanced dataset consisting of Yeast proteins which locate in 22 subcellular locations; some of them have multiple labels. The prediction performance evaluated in three measures has proven the usefulness of the integrated information and the advance of the fuzzy k-NN classifier.

Since each protein subcellular localization prediction approaches has its own limitations and merits, there is a trend to integrate all available methods in order to build practical completed prediction systems. Therefore, our proposed method can be integrated in such systems to complement the other available prediction methods to enhance the overall prediction performance.

## Methods

### Feature extraction

#### GO annotation

Gene Ontology Annotation (GOA) database [30] which includes GO annotation for non-redundant proteins from many species in the UniProtKB/Swiss-Prot database [29] is a useful resource to mine informative features for prediction of protein subcellular localization. This study also applied GOA to build prediction features. The GOA database was directly downloaded from [31].

Each entry in the GOA represents a mapping from a protein in the UniProtKB/Swiss-Prot database to a GO term. One protein might be mapped to multiple GO terms, it implies the biological reality that a particular protein may function in several process, contain domains that carry out diverse molecular functions, and anticipate in multiple alternative interaction with other proteins, organelles, or locations in the cell. Thus, by featuring a protein by GO terms one can expect to capture its essential characteristics which are informative to predict localization.

However one should notice that the assignment of GO terms to proteins is not an automatic process. For example, some GO terms are manually assigned after experts read all PUBMED abstracts or full papers which refer to the proteins. Such manually assignment should not be utilized for training an automatic computational prediction system. Therefore, in this study we remove all mappings that are manually curated. For example, the protein P38858 located in nucleus compartment is assigned with the following GO terms: GO:0005634, GO:0005737, GO:0006098, GO:0009051, GO:0016787 and GO:0017057. The GO terms GO:0005634 "nucleus" which was assigned manually by analysing PUBMED references is clearly an obvious localization indicator. Among 6 GO terms, we select only two of them (GO:0005634 and GO:0017057) which are automatically assigned to represent as the protein prediction features.

#### Incorporating neighborhood information

Probabilistic functional gene networks are powerful theoretical frameworks for integrating heterogeneous functional genomics and proteomics data into objective models of cellular systems. They can be utilized to generate testable hypotheses regarding specific gene functions and associations. In this study, we use such a network to grasp functional relations which proteins might have in order to enrich the protein subcellular location prediction features. YeastNet [32] is constructed by integrating ten different biological data sources such as protein interaction databases, mRNA co-expression, co-citation evidence, genetic interactions. It has 5,483 nodes corresponding to Yeast proteins and 10.2803 edges representing probabilistic functional relations. Each edge in the network is associated with a weight value indicating the reliability of the relation. The network is available to download from [33].

The process of adding neighborhood information to build features for subcellular localization prediction can be explained in following steps:

1) Given a protein *p*, find set of proteins which connect to *p *in YeastNet and their connection reliable weight must pass a threshold value *c*. From this set we selected top *k *proteins {*p*_1_, *p*_2 _.. *p*_*k*_} whose connections reliable weights are highest. In this study, *k *is set to 5 and *c *is set to 2.5.

2) For each protein in the set {*p*, *p*_1 _.. *p*_*k*_} find the set of associate GO terms. Let *G*(*p*) *G*(*p*_1_),.. *G*(*p*_*k*_) are those sets.

3) The set of GO terms which are considered associated to the protein *p *after incorporating the neighborhood information is defined as:

(7) $$\tilde{\tilde{G}}(p\text{) = }G(p\text{)}\cup G(p_{1}\text{)}\cup G(p_{2}\text{)}\cup ..G(p_{k}\text{)}$$

Although the total number of GO terms in database is large, only 1200 GO terms are found in Yeast proteins. Let {*GO*_1_, *GO*_2_,.. *GO*_1200_} be the set of those GO terms, using this set we create a space, so called GO-space, on which we can define a vector P to represent the protein features as:

(8) *P *= [*g*_1 _*g*_2 _... *g*_1200_]

Where

(9) $$g_{i}=\{\begin{array}{ll} 1 & \text{if }GO_{i}\subset \tilde{\tilde{G}}(p)\\ 0 & \text{otherwise} \end{array}\;$$

#### Amino acid and dipeptide compositions

Although integrating neighborhood information can increase the prediction coverage, there are still proteins which can not be represented by GO-based features. To handle such proteins we use amino acid compositions and dipeptide compositions information to predict their location labels. Many previous methods for predicting protein subcellular localization were replied on those features.

Given a protein sequence, the composition of an amino acid *a*_*i *_(*i *= 1.. 20) is estimated as:

(10) $$f(a_{i}\text{) = }\frac{\text{number of }a_{i}\text{ ocurrences}}{\text{total number of amino acids in protein}}$$

The composition of a dipeptide is estimated as:

(11) $$f(a_{i}a_{j}\text{) = }\frac{\text{number of }a_{i}a_{j}\text{occurrences}}{\text{total number of posible dipeptides in protein}}$$

There are 20 amino acids, thus each protein can be represented by a 420-dmension vector consisting of 20 features from amino acid composition and 400 features from dipeptide compositions

### Fuzzy k-NN classification

The k-nearest neighbor (k-NN) rule is one of the oldest and simplest methods for performing nonparametric classification. The main idea of k-NN can be stated as following: given a test sample with unknown label, its label is assigned according to the labels of its *k *nearest neighbors in the training set.

Fuzzy k-NN classification method is a special variation of the k-NN classification family. Instead of roughly assigning labels by a voting on labels of nearest neighbors, it attempts to estimate membership values which indicates how much degree the query sample belong to classes. In the context of our prediction problem where some proteins can be classified to multiple labels, such fuzzy logic theory approach is thus especially appropriate.

Let {*P*_1_, *P*_2_,.., *P*_*N*_} be the set of vectors representing N proteins in the training set which has been classified to categories *c*_1_, *c*_2_,.. *c*_22_, let *P *be a protein to be classified. In the first step, the fuzzy k-NN assigns membership values for each protein in the training set to different categories. For the single-label classification case, the simplest method is to set the membership value *v*_*c*_(*P*_*i*_) of a protein *P*_*i *_respecting to class *c *to 1 if the real label of *P*_*i *_is *c*, otherwise, set it to zero. This can be generalized for the multi-label classification case as follow:

(12) $$v_{c}(P_{i})=\{\begin{array}{cc} 1/\left|L(P_{i})\right| & \text{if }c\text{ belongs to }L(P_{i})\\ 0 & \text{if }c\text{ does not belong to }L(P_{i}) \end{array}$$

where *L*(*P*_*i*_) denote the set of locations that protein *P*_*i *_exist in.

After initializing membership values for all proteins in the training data, membership value of *P *to class *c *is determined by combining the membership values of its neighbors, taking into account their closeness to the protein under consideration. The closeness between any two proteins is defined through some distance measures. Let's define *k *be the number of nearest neighbors which are interested for estimating the membership value of *P*, let *P*^(*j*) ^be the *j*^*th *^nearest neighbor of *P *in the training set, *d*(*P*, *P*^(*j*)^) be the distance between *P *and its *j*^*th *^nearest neighbor. A fuzzy strength parameter *m *is defined (*m *> 1) to determine how heavily the distance is weighted when calculating each nearest neighbor's contribution to the membership value. The membership value of *P *to class *c *is then calculated as following equation:

(13) $$u_{c}(P)=\frac{\sum_{j=1}^{k}v_{c}(P^{\left(j\right)})d{\left(P,P^{\left(j\right)}\right)}^{2/(1-m)}}{\sum_{j=1}^{k}d{\left(P,P^{\left(j\right)}\right)}^{2/(1-m)}}$$

And finally, the protein *P *is classified as classes to which the membership values of *P *are highest. The number of topmost classes is decided by user.

#### Distance measures

In the proposed method, two classifiers are used to predict subcellular locations. If a protein can be features in GO-space, its locations will be determined by the Fuzzy k-NN – GO classifier. Otherwise, the amino acid composition and dipeptide compositions are estimated then Fuzzy k-NN – Comp classifier will assign localization labels for the query protein. In Fuzzy k-NN GO classifier, the distance between two proteins represented in GO-space is estimated as:

(14) $$\begin{array}{l} d(P,P^{\left(j\right)})=1-\frac{P.P^{\left(j\right)}}{\left\|P\right\|\times \left\|P^{\left(j\right)}\right\|}\\ \text{where }P.P(j)\text{ is the dot product of vectors }P\text{ and }\\ P(j)\text{ and }\left\|P\right\|\text{ and }\left\|P^{\left(j\right)}\right\|\text{ are their moduli}\text{.} \end{array}$$

On the other hand, in Fuzzy k-NN Comp, the distance between two proteins is defined by Euclidian distance measure.

## Competing interests

The authors declare that they have no competing interests.

## Authors' contributions

TQT conceptualized the project, implemented and validate the method. DL helped with writing the manuscript and provided many valuable suggestions directing the study. All authors read and approved the final manuscript.
